# Association between work shifts, occupational stress, and abdominal obesity in female workers in Southern Brazil

**DOI:** 10.3389/fpubh.2025.1705335

**Published:** 2025-11-18

**Authors:** Harrison Canabarro Arruda, Anderson Garcez, Janaína Cristina da Silva, Ingrid Stähler Kohl, Heloísa Theodoro, Raquel Canuto, Maria Teresa Anselmo Olinto

**Affiliations:** 1 Post-Graduate Program in Food, Nutrition and Health, Faculty of Medicine, Federal University of Rio Grande do Sul, UFRGS, Porto Alegre, Brazil; 2 Post-Graduate Program in Medical Sciences: Endocrinology, Federal University of Rio Grande do Sul State, UFRGS, Porto Alegre, Brazil; 3 Post-Graduate Program in Collective Health, University of Vale do Rio dos Sinos, Unisinos, São Leopoldo, Brazil; 4 Post-Graduated Program in Department of Nutrition, University of Caxias do Sul, UCS, Caxias do Sul, Brazil

**Keywords:** occupational stress, shift work, abdominal obesity, women, cross-sectional repeated survey

## Abstract

**Background:**

Night work and occupational stress contribute to weight gain and abdominal fat accumulation through behavioral and metabolic changes. This study investigated the relationship between occupational stress, work shifts, and abdominal obesity among female workers in Southern Brazil.

**Methods:**

This repeated cross-sectional study included two samples collected in 2017 and 2022, each comprising 400 female workers from two factories located in Southern Brazil. Abdominal obesity was measured by waist circumference (WC ≥ 88 cm), and occupational stress was assessed using the Job Stress Scale - short version. Data on demographic, socioeconomic, occupational, reproductive, and health variables were collected. Poisson regression with robust variance was used for multivariate analysis, stratified by occupational stress (absence vs. presence) and adjusted for potential confounders.

**Results:**

The workers’ mean ages were 35.8 ± 9.0 years (2017) and 34.2 ± 9.9 years (2022). The prevalence of abdominal obesity was 45.1% (95% confidence interval [CI]; 40.2–50.0) in 2017 and 43.0% (95% CI, 38.1–47.9) in 2022, while occupational stress was observed in 22.9% (95% CI, 18.8–27.1) and 21.0% (95% CI, 17.0–25.0) of the workers, respectively. Among workers with occupational stress, nightshift workers were twice more likely to have abdominal obesity compared to daytime workers (2017; prevalence ratio [PR] = 2.23, 95% CI; 1.47–3.38, *p* < 0.001; 2022; PR = 1.80, 95% CI; 1.06–3.06, *p* = 0.029). No significant association was observed between work shifts and abdominal obesity among workers without occupational stress.

**Conclusion:**

Occupational stress significantly modified the relationship between nighttime work and abdominal obesity. This study found a high prevalence of abdominal obesity, especially among female night shift workers, with no significant changes in prevalence rates between 2017 and 2022.

## 1 Introduction

Global data show a prevalence of approximately 41.5% of abdominal obesity, with recently increasing rates and a higher prevalence in women than in men (1). According to the National Health Survey, the prevalence of abdominal obesity in Brazilian women was estimated at 52.1% in 2013 (2). In Southern Brazil, the prevalence of abdominal obesity among adult women (20–60 years) significantly increased from 23.3 to 46.9% between 2005 and 2015 (3). Abdominal obesity is a multifactorial disorder influenced by occupational (work shift and occupational stress), socioeconomic (education and income), behavioral (physical activity and diet), biological (sex), and genetic factors (4, 5). It is a significant risk factor for cardiovascular diseases, type II diabetes, metabolic syndrome, dyslipidemia, breast cancer, and increased all-cause mortality (1–3, 6).

Shift work aims to balance service provision, worker well-being, and sector productivity (7) and organizes teams for day, evening, and night shifts, ensuring 24-h service continuity (7, 8). However, this system has been shown to harm workers’ health, contributing to sleep disorders (9), increased cardiovascular disease risk (10), and metabolic changes that promote obesity, particularly abdominal obesity (11). Nighttime shifts have been extensively studied for their potential health risks to workers (12). Night work involves labor duties from 12–5 a.m. (13). Exposure to night shifts affects various behaviors and health factors that contribute to visceral fat accumulation, including poor diet quality, reduced meal frequency, physical inactivity, and sleep restriction (14–17). A meta-analysis of nine studies found a strong association between shift work and abdominal obesity, with a 35% higher likelihood of abdominal obesity among night shift workers than day shift workers (11).

The International Labour Organization (ILO) defines occupational stress as an emotional response to the mismatch between job demands and the worker’s ability to manage these demands, based on available resources and skills (18). Globally, the prevalence of occupational stress varies widely, ranging from 20.5–78.4% (19–21). In Brazil, previous data showed a prevalence of 46.5% among 539 female workers at a poultry processing plant in Southern Brazil (22), and 23.8% among 290 nutritionists at a university hospital in Southeastern Brazil (23). Additionally, women are often more susceptible to occupational stress because of dual work shifts, balancing work and domestic responsibilities (23).

Previous studies suggest an association between occupational stress and work shifts (19), particularly a higher occurrence of stress among night shift workers (19, 24), possibly due to reduced ability to manage the negative effects of demand/control imbalance (25). Similarly, occupational stress has been linked to abdominal obesity (26, 27); However, this association remains inconsistent, as the relationship between shift work, abdominal obesity, and occupational stress is still not well understood—particularly concerning the role of occupational stress as a potential mediator in the association between work shifts and abdominal obesity. Moreover, the necessity of conducting this study is further underscored by the scarcity of research involving samples of female blue-collar workers, given that previous studies have predominantly focused on healthcare and white-collar professionals, often using mixed-sex samples (28, 29). Thus, the main purpose of this study was to examine the relationship between occupational stress, work shifts, and abdominal obesity using two samples of female workers from Southern Brazil, collected in 2017 and 2022. We hypothesize that occupational factors play a significant role in influencing metabolic health among female shift workers.

## 2 Methods

### 2.1 Study design and population

This repeated cross-sectional study included two occupation-based samples comprising adult female workers from two factories within the same business group in Southern Brazil. Both samples were collected at different time points (2017 and 2022) using similar methodologies to ensure the comparability of results. This investigation is part of a larger research project entitled, Health Conditions of Female Shift Workers: A Longitudinal Occupational Health Study / “Condições de saúde de mulheres trabalhadoras de turnos: estudo longitudinal de saúde ocupacional (ELO SAÚDE).” This research is based on primary data collected by our research group with the aim of investigating a wide range of health conditions among female shift workers. The study was reviewed and approved by the Research Ethics Committee of the University of Vale do Rio dos Sinos, and all participants provided informed consent.

### 2.2 Samples and sampling

For both samples (2017 and 2022), all female workers aged ≥18 years from two factories belonging to the same business group specializing in household plastic products were considered eligible. In 2017, only production-sector workers were included, whereas in 2022, workers from both the production and administrative sectors (*n* = 93) were included. The exclusion criteria were pregnancy, temporary absence from work, and employment in the same work shift for less than 3 months.

In 2017, 450 out of 553 eligible workers were interviewed after accounting for losses and refusals (30–32). In 2022, 452 out of 546 eligible workers were interviewed, among whom 102 had also participated in the 2017 assessment. To ensure independence between the two samples, these 102 repeated respondents were not included simultaneously in both datasets. Instead, they were randomly allocated to one of the two samples, with 51 assigned to the 2017 dataset and 51 to the 2022 dataset. Following this procedure, each sample consisted of 400 unique participants. A sensitivity analysis was performed, which demonstrated that the distribution of sample characteristics remained consistent across the 2017 and 2022 samples after the random allocation of repeated respondents.

### 2.3 Data collection and instruments

Data were collected from June to August 2017 (first study) and August 2022 to February 2023 (second study). In both studies, a standardized, precoded, and pretested questionnaire was administered through face-to-face workplace interviews, covering demographic, socioeconomic, reproductive, behavioral, health, and anthropometric characteristics. Interviewers in each study participated in a training program. Before fieldwork, a pilot study with 21 interviews was conducted. Fieldwork quality control included interviewer supervision and data verification by telephone for 10% of the sample, with some questions reapplied to ensure response consistency. Data coding was performed by interviewers and supervised by the research coordinator.

### 2.4 Occupational stress

Occupational stress was assessed using the Job Stress Scale adapted and validated in Brazilian Portuguese (33), comprising 17 questions, each scored from 1 to 4 and divided into four domains; demand, intellectual discretion, authority, and social support. The control domain was calculated by summing intellectual discretion and authority scores. The combined demand and control scores were categorized into “high” and “low,” based on the median cutoff in each sample. Occupational stress was defined as the combination of high demand and low control according to Karasek’s job strain model (33, 34).

### 2.5 Work shift

The companies’ work shifts included three fixed shifts, meaning all workers consistently worked the same shift; morning (06:00 a.m.–02:00 p.m.), afternoon (02:00 p.m.–10:00 p.m.), and night (10:00 p.m.–07:00 a.m.). Additionally, women working administrative hours (08:00 a.m.–05:00 p.m.) were included. Work shifts were categorized based on the starting time into day shifts (06:00 a.m.–02:00 p.m.) and night shifts (10:00 p.m.–06:00 a.m.).

### 2.6 Abdominal obesity (outcome)

Abdominal obesity was assessed by measuring waist circumference (WC) using a non-extensible tape with 1 mm accuracy, placed directly on the skin at the midpoint between the last costal arch and iliac crest. Two measurements were taken for each woman, and the average was used to determine the outcome (35). Women with a WC of ≥ 88 cm were considered to have abdominal obesity (35).

### 2.7 Covariates

Multivariable analysis determined the demographic, socioeconomic, behavioral, reproductive, and health characteristics to describe the samples and control for potential confounding factors. The same approach was used to collect all variables in 2017 and 2022. Demographic characteristics included age groups (18–30, 31–40, and > 40 years), self-reported skin color (white and others [brown, black, indigenous, yellow/oriental]), and marital status (with or without a partner). Socioeconomic characteristics included per capita family income (< 1 minimum wage, 1–2 minimum wages, > 2 minimum wages); calculated as the sum of the reported income of each family member in the previous month divided by the number of household residents and categorized by the Brazilian national minimum wage (2017 = BRL 937.00, 2022 = BRL 1212.00) and years of education (≤ 8, 9–11, > 11 years). Behavioral characteristics included leisure-time physical activity (assessed by the question, “In the last week, did you engage in any physical activity for leisure, sports, or exercise, excluding commuting?”), self-reported sleep quality (measured by the question “During the last month, how would you rate your sleep overall?” [very good/good or poor/very poor]), or current smoking status (never smoked, former smoker, or current smoker). Reproductive and health characteristics included parity (determined by the number of reported pregnancies and classified as nulliparous, primiparous, or multiparous) and self-perceived health (reported on a five-point Likert scale [excellent, very good, good, fair, or poor]).

### 2.8 Statistical analysis

The collected data were entered using a double-entry procedure in the EpiData program (Centers for Disease Control and Prevention, Atlanta, GA, USA), including a comparison of entries and consistency analysis. Descriptive statistics were used to describe the distribution of the sample characteristics, exposures, outcomes, and abdominal obesity using absolute and relative frequencies. To compare the distribution of abdominal obesity and occupational stress prevalence according to the characteristics in the 2017 and 2022 samples, Chi-square test was used for heterogeneity of proportions or linear trends.

In the multivariate analysis, Poisson regression with robust variance was used to estimate unadjusted and adjusted prevalence ratios (PR) for associations between occupational stress, work shift, and abdominal obesity, with 95% confidence intervals (95% CI). The following multivariate adjustment models were considered; models I (unadjusted analysis), II (adjusted for demographic and socioeconomic characteristics), and III (adjusted for model II characteristics plus behavioral, reproductive, and health characteristics). All variables associated with abdominal obesity and/or occupational stress (*p*-value < 0.20 in the unadjusted analysis) were considered potential confounders and included in the multivariate analysis based on the conceptual model defined *a priori* (36). Stratified analyses were conducted to verify the interaction between occupational stress and work shift concerning abdominal obesity using the Mantel–Haenszel test. A complementary analysis was conducted to assess potential changes in the investigated associations after excluding administrative sector workers, and no significant differences were observed.

All analyses were stratified by study year (2017 and 2022) and conducted using Stata version 14 (StataCorp LP, College Station, Texas, USA), with a significance level of 5%.

## 3 Results

The analysis included 397 workers with a mean age of 35.8 ± 9.0 years (2017) and 400 workers with a mean age of 34.2 ± 9.9 years (2022). Three workers in 2017 were excluded owing to incomplete data on abdominal obesity and/or occupational stress.

Table 1 presents the general characteristics of the 2017 and 2022 samples. In both studies, most workers were white (67.4% in 2017 and 70.2% in 2022), earned 1–2 minimum wages (52.6% in 2017 and 40.3% in 2022), were physically inactive (76.3% in 2017 and 70.8% in 2022), reported good sleep quality (70.3% in 2017 and 67.3% in 2022), had never smoked (74.6% in 2017 and 77% in 2022), worked the day shift (76.8% in 2017 and 79.7% in 2022), and had no occupational stress (77.1% in 2017 and 79.0% in 2022). Compared to 2017, the 2022 sample included a higher proportion of young workers (18–30 years; 32.7% vs. 40%), workers with higher education levels (>11 years; 8.8% vs. 41.7%), and workers without partners (44.6% vs. 52.8%).

**Table 1:** General characteristics of the samples and prevalence of abdominal obesity according to the characteristics of female workers from Southern Brazil, 2017 and 2022.

Characteristics	2017 *n* (%)	2022 *n* (%)	2017		2022	
			*n* (%)	*p*-value	*n* (%)	*p*-value
Total	397 (100)	400 (100)	179 (45.1)		172 (43.0)	
Age (years)				<0.001^b^		<0.001^b^
18–30	130 (32.7)	160 (40.0)	39 (30.0)		40 (25.0)	
31–40	137 (34.6)	132 (33.0)	69 (50.7)		69 (52.3)	
> 40	130 (32.7)	108 (27.0)	71 (54.6)		63 (58.3)	
Skin color	*n* = 396	*n* = 392		0.220^a^		0.970^a^
White	267 (67.4)	281 (70.2)	115 (43.1)		121 (43.1)	
Other	129 (32.6)	119 (29.8)	64 (49.6)		51 (42.9)	
Marital status				0.017^a^		<0.001^a^
Without partner	177 (44.6)	211 (52.8)	68 (38.4)		73 (34.6)	
With partner	220 (55.4)	189 (47.2)	111 (50.5)		99 (52.4)	
Per capita family income (minimum wages)				0.500^b^		0.016^b^
< 1	144 (36.3)	155 (38.7)	60 (41.7)		79 (51.0)	
1–2	209 (52.6)	161 (40.3)	100 (47.8)		66 (41.0)	
> 2	44 (11.1)	84 (21.0)	19 (43.2)		27 (32.1)	
Education (years of study)				0.034^b^		0.003^b^
≤ 8	59 (14.9)	25 (6.3)	33 (55.9)		15 (60.0)	
9–11	303 (76.3)	209 (52.3)	134 (44.2)		99 (47.5)	
> 11	35 (8.8)	166 (41.7)	12 (34.3)		58 (34.9)	
Leisure-time physical activity				0.014^a^		0.039^a^
No	303 (76.3)	283 (70.8)	147 (48.5)		131 (46.3)	
Yes	94 (23.7)	117 (29.2)	32 (34.0)		41 (35.0)	
Sleep quality				0.009^a^		0.173^a^
Very good/good	279 (70.3)	269 (67.3)	114 (40.9)		122 (45.4)	
Poor/very poor	118 (29.7)	131 (32.7)	65 (55.1)		50 (38.2)	
Current smoking status				0.009^a^		0.841^a^
Never smoked	296 (74.6)	308 (77.0)	132 (44.6)		130 (42.2)	
Former smoker	71 (17.9)	66 (16.5)	40 (56.3)		30 (45.5)	
Current smoker	30 (7.6)	26 (6.5)	7 (23.3)		12 (46.2)	
Parity				0.005^b^		<0.001^b^
Nulliparous	117 (29.5)	156 (39.0)	38 (32.5)		46 (29.5)	
Primiparous	130 (32.7)	113 (28.2)	66 (50.8)		56 (49.6)	
Multiparous	150 (37.8)	131 (32.8)	75 (50.0)		70 (53.4)	
Self-perceived health				0.001^b^		0.012^a^
Excellent/very good	91 (22.9)	110 (27.5)	28 (30.8)		36 (32.7)	
Good	184 (46.4)	186 (46.5)	81 (44.0)		81 (43.5)	
Fair/poor	122 (30.7)	104 (26.0)	70 (57.4)		55 (52.9)	
Work shift				0.003^a^		0.022^a^
Day shift	305 (76.8)	319 (79.7)	125 (41.0)		128 (40.3)	
Night shift	92 (23.2)	81 (20.3)	54 (58.7)		44 (54.3)	
Occupational stress				0.636^a^		0.204^a^
Absence	306 (77.1)	316 (79.0)	136 (44.4)		141 (44.6)	
Presence	91 (22.9)	84 (21.0)	43 (47.2)		31 (36.9)	

a Pearson’s Chi-Square test for heterogeneity proportion.

b Test for linear trend.

The prevalence of abdominal obesity was similar between both studies; 45.1% (95% CI; 40.2–50.0) in 2017 and 43.0% (95% CI; 38.1–47.9) in 2022 (Chi-squared *p* = 0.396). Similar trends were observed for occupational stress (22.9%; 95% CI; 18.8–27.1 in 2017 and 21.0%; 95% CI; 17.0–25.0 in 2022; Chi-squared *p* = 0.549) and night shift work (23.2%; 95% CI; 19.0–27.3 in 2017 and 20.3%; 95% CI; 16.3%–24.2 in 2022; Chi-squared *p* = 0.345).

Table 1 shows the prevalence of abdominal obesity according to the worker characteristics in 2017 and 2022. The highest prevalence of abdominal obesity was observed in older workers, those living with partners, those with lower educational levels, those not engaged in physical activity, those with ≥ 1 pregnancies, and those with poor self-perceived health. In 2017, poor sleep quality and smoking were associated with a high prevalence of abdominal obesity. By 2022, an inverse relationship between income and abdominal obesity was observed, with the highest prevalence among workers earning < 1 minimum wage. Night shift work was associated with a higher prevalence of abdominal obesity in 2017 and 2022, whereas occupational stress was not significantly associated with abdominal obesity.

Interaction analysis between work shifts, occupational stress, and abdominal obesity revealed a significant interaction of occupational stress (absence vs. presence) on the association between work shifts and abdominal obesity in 2017 (*p* = 0.003) and 2022 (*p* = 0.014). No interaction was observed between work shifts and occupational stress relating to abdominal obesity (*p* = 0.570 [2017] and *p* = 0.135 [2022]). In 2022, occupational stress was associated with work shifts in the bivariate analysis (data not shown).

Table 2 presents the unadjusted and adjusted prevalence ratios for the association between work shifts and abdominal obesity for the total 2017 and 2022 samples and samples stratified by the presence or absence of occupational stress. Multivariate analysis showed that night shift workers had a 43 and 35% higher probability of abdominal obesity than day-shift workers in 2017 and 2022, respectively. This effect was observed only among workers with occupational stress (2017; PR = 2.23, 95% CI; 1.47–3.38, *p* < 0.001 and 2022; PR = 1.80, 95% CI; 1.06–3.06, *p* = 0.029). Among the workers without occupational stress, no significant association was observed between work shifts and abdominal obesity.

**Table 2:** Unadjusted and adjusted Prevalence Ratios (PR) for the association between work shift and abdominal obesity in the total sample and according to the absence and presence of occupational stress in female workers from Southern Brazil, 2017 and 2022.

Work shift	Abdominal obesity (WC ≥ 88 cm)	Model I		Model II		Model III	
	*n* (%)	PR (95% CI)	*p*-value*	PR (95% CI)	*p*-value*	PR (95% CI)	*p*-value*
2017							
Total (*n* = 397)							
Work shift			0.001		0.004		0.003
Day shift	125 (41.0)	1.00 (ref.)		1.00 (ref.)		1.00 (ref.)	
Night shift	54 (58.7)	1.43 (1.15–1.78)		1.38 (1.11–1.72)		1.39 (1.11–1.73)	
Without occupational stress (*n* = 306)							
Work shift			0.063		0.155		0.165
Day shift	97 (41.6)	1.00 (ref.)		1.00 (ref.)		1.00 (ref.)	
Night shift	39 (53.4)	1.28 (0.99–1.67)		1.22 (0.92–1.59)		1.21 (0.92–1.58)	
With occupational stress (*n* = 91)**							
Work shift			<0.001		0.001		<0.001
Day shift	28 (38.9)	1.00 (ref.)		1.00 (ref.)		1.00 (ref.)	
Night shift	15 (78.9)	2.03 (1.40–2.95)		1.95 (1.33–2.85)		2.23 (1.47–3.38)	
2022							
Total (*n* = 399)							
Work shift			0.014		0.128		0.083
Day shift	128 (40.2)	1.00 (ref.)		1.00 (ref.)		1.00 (ref.)	
Night shift	44 (54.3)	1.35 (1.06–1.72)		1.19 (0.95–1.50)		1.22 (0.97–1.54)	
Without occupational stress (*n* = 316)							
Work shift			0.212		0.499		0.362
Day shift	112 (43.1)	1.00 (ref.)		1.00 (ref.)		1.00 (ref.)	
Night shift	29 (51.8)	1.20 (0.90–1.61)		1.10 (0.83–1.46)		1.14 (0.86–1.51)	
With occupational stress (*n* = 84)**							
Work shift			0.003		0.017		0.029
Day shift	16 (27.1)	1.00 (ref.)		1.00 (ref.)		1.00 (ref.)	
Night shift	15 (60.0)	2.21 (1.30–3.75)		1.86 (1.11–3.11)		1.80 (1.06–3.06)	

*p-value for the Wald test for heterogeneity of proportions obtained by Poisson regression with robust variance.

**p = 0.003 (2017) and *p* = 0.014 (2022) for Mantel–Haenszel test: test of interaction of occupational stress (absence vs. presence) for the association between work shift and abdominal obesity.

Model I: unadjusted (crude analysis); Model II: adjusted for sociodemographic characteristics (age, marital status, family income, and education); Model III: adjusted for Model I + behavioral, reproductive, and health characteristics (leisure-time physical activity, sleep quality, and parity).

## 4 Discussion

This study examined the relationship between occupational stress, work shifts, and abdominal obesity among female workers in Southern Brazil in 2017 and 2022. Occupational stress was found to modify the relationship between work shifts and abdominal obesity, with women under occupational stress and working night shifts being approximately twice more likely to have abdominal obesity than those with occupational stress but working day shifts. Despite the lack of significant differences between both periods, the prevalence of abdominal obesity remained high, particularly among female night shift workers.

Previous studies have highlighted the link between work shifts and weight gain, identifying night shifts as a risk factor for abdominal obesity (11, 37, 38). A cross-sectional study of 200 workers at a university hospital in Southern Brazil showed that night shift workers had a 193% higher likelihood (odds ratio [OR]; 2.93, 95% CI; 1.57–5.46) of having abdominal obesity than day shift workers (39). Similarly, in the same region, a study involving 1,206 poultry processing workers found a 45% increased likelihood (OR; 1.45, 95% CI; 1.10–1.92) of abdominal obesity among night shift workers (37). Additionally, an analysis of data from the 2013 National Health Survey in Brazil revealed a 27% higher risk (OR; 1.27, 95% CI; 1.10–1.46) of abdominal obesity among night shift workers than day shift workers (40). These findings align with the literature, reinforcing the association between night shift work and abdominal obesity and highlighting the physiological and psychosocial risks posed by night shifts (11, 37, 39).

Moreover, the literature discussed the relationship between night shift work and occupational stress (22, 25, 41, 42). A cross-sectional study of 654 nurses found that night shift workers had a 116% higher risk (OR; 2.16, 95% CI; 1.03–4.66) of experiencing occupational stress, according to the Effort-Reward Imbalance Model (41). Conversely, an observational study involving 124 nursing professionals, using the Job Stress Scale, found no significant association between occupational stress and work-related variables after adjustment (42). Similarly, a previous study involving 539 female industrial workers found no association between work shift and perceived stress, as measured by the Perceived Stress Scale (22). The discrepancies among these findings may be attributed to the use of different tools for assessing occupational stress and variations in the nature of the work among the samples. This study found an association between occupational stress and night work in 2022. A supplementary analysis revealed that night shift workers scored proportionally lower in the control domain (intellectual discretion + authority) and social support domain than day shift workers.

Night shift work is a known inducer of circadian misalignment, which is a condition that results from the disruption of regular sleep–wake cycles. This misalignment leads to alterations in the balance of appetite-stimulating and satiety hormones, ghrelin and leptin, respectively. Such hormonal disruptions can affect meal frequency and the overall energy balance, contributing to weight gain (15, 43). Additionally, night shift work can negatively impact sleep patterns and reduce opportunities for physical activity during the day, further promoting weight gain (40, 44).

**Figure 1:**
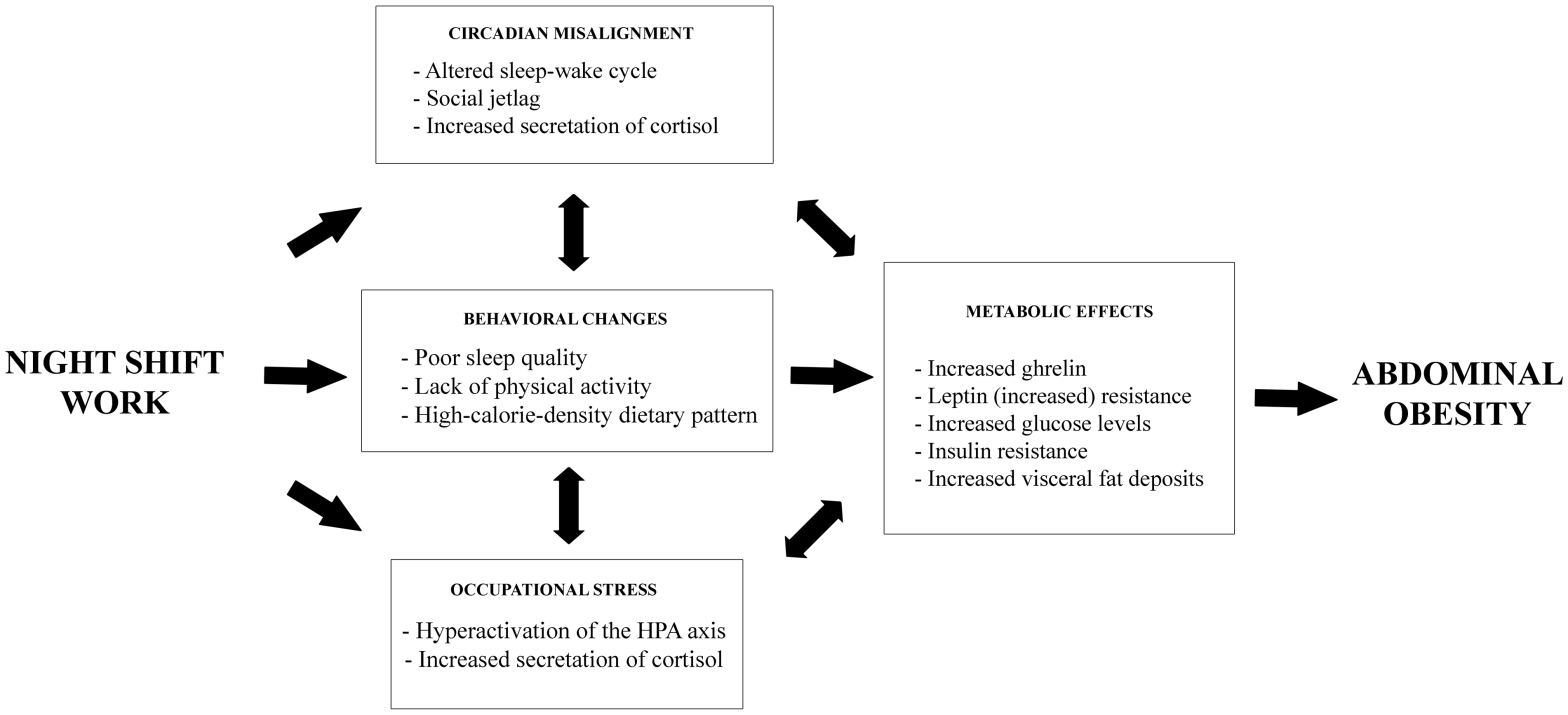
Schematic model of health consequences related to night work and occupational stress for the development of abdominal obesity.

The hypothesis that occupational stress induces abdominal obesity is supported by endocrine and behavioral mechanisms (25). The endocrine pathway involves hyperactivation of the hypothalamic–pituitary–adrenal axis, resulting in excessive cortisol secretion. Cortisol, a hormone that significantly contributes to fat accumulation, particularly in the abdominal region, promotes insulin resistance, creating a feedback loop that contributes to abdominal obesity (25). Behaviorally, individuals with occupational stress prefer foods with higher caloric density and palatability (22, 25), have reduced leisure-time physical activities (25, 45), and have shorten sleep duration (25, 46). These behavioral changes mirror the effects of shift work, providing a rationale for investigating the interaction between occupational stress and shift work in the current study. Figure 1 illustrates the pathways through which night work and occupational stress contribute to the development of abdominal obesity.

Despite the observed increase in the prevalence of excess adiposity in the general population in 2017 and 2022 (47), this study found similar prevalence rates of abdominal obesity in both samples. This could be attributed to the higher proportion of young workers (aged 18–30 years) and those with higher educational levels in the 2022 sample, as both factors were associated with a lower prevalence of abdominal obesity. Additionally, the high turnover rate among workers may have contributed to the healthy worker effect, suggesting that those who remained employed with the company over the five-year period were less susceptible to developing abdominal obesity.

Here, the prevalence of abdominal obesity was similar to that found in a population-based study of women in Southern Brazil, which reported a prevalence of 46.9% (48). However, other studies involving working female populations have shown different values, with prevalence rates of 31.6% in Poland and 73.2% in Iran (49, 50). Specifically, among night shift workers, our study reported prevalence rates of 58.3% in 2017 and 54.3% in 2022. A longitudinal study of hospital workers in Northeastern Brazil demonstrated a significant increase in the prevalence of abdominal obesity, from 53.2% in 2019 to 60.6% in 2020 (51). The prevalence of occupational stress found in our sample was consistent with previous findings in women using tools based on the Job Content Questionnaire (52, 53), although prevalence varied from 20.5–46.5% in considering other assessment tools, such as the Effort-Reward Model and Perceived Stress Scale (19, 22, 41). A longitudinal study of hospital workers in Northeastern Brazil found a prevalence of 14.2% in 2019, which increased to 29.4% in 2020 (Job Stress Scale) (51). Conversely, a longitudinal study of hospital workers in Australia found an occupational stress prevalence of 24.3% in 2020 and 25.4% in 2021, with no significant difference (Perceived Stress Scale) (54). Overall, these comparisons highlight the consistency of our findings with previous research and reinforce that similar associations have been observed across diverse populations and occupational settings.

This study is among the first to explore the associations between work-related stress, work shifts, and abdominal obesity in adult female workers. Among the strengths of this study was the process of obtaining two independent samples from cross-sectional occupation-based studies conducted 5 years apart, involving women from the same business group. The use of consistent methodological procedures aimed to ensure uniformity in data collection, thereby making the results comparable. The instruments used, including the measurement of WC and application of a previously established cutoff point to define abdominal obesity in adult women, were validated before the study (35). Additionally, the Job Stress Scale – short version was adapted and validated for Brazilian-Portuguese (33). Importantly, this study accounted for significant confounding factors in the multivariable analysis and examined occupational stress as an interaction factor in the relationship between work shift and abdominal obesity. However, these findings should be interpreted with caution because of several limitations. First, the study was conducted on working female populations from companies located in a single city in Southern Brazil, limiting the generalizability of the results to other populations. Specifically, the target population included workers in fixed-shift regimes; therefore, the findings may not apply to workers in rotating shifts or other work schedules. Second, as a cross-sectional study, it is susceptible to reverse causality because exposures and outcomes were assessed simultaneously, thereby preventing causal inference. For instance, the study did not determine the duration of occupational stress exposure. Nevertheless, reverse causality is unlikely provided the biological plausibility of the pathophysiological mechanisms linking occupational stress and abdominal obesity in night workers. Further research is required to confirm these findings. Third, although WC was measured between expiration and inspiration phases, fasting status was not controlled; however, measurements were taken during work hours, distant from meal times, minimizing potential bias. Fourth, using a single question to assess leisure-time physical activity and sleep quality may have limited measurement precision and introduced residual confounding in the analysis of abdominal obesity. Finally, although a complementary analysis showed that including administrative workers did not change the main results, their small number limited the possibility of adjusted or stratified analyses. This may represent a potential limitation, given the distinct caloric expenditure and occupational stress levels associated with these positions.

In conclusion, this study identified a significant relationship among occupational stress, work shifts, and abdominal obesity, with occupational stress as a key effect modifier. Night shift workers experiencing occupational stress were approximately twice more likely to have abdominal obesity than day shift workers. Between 2017 and 2022, no significant differences were observed in the prevalence of occupational stress, night shift work, and abdominal obesity. Targeted interventions within the occupational environment, especially for night shift female workers, are essential for preventing occupational stress, which could reduce the incidence of abdominal obesity. Both conditions share similar pathophysiological mechanisms and behavioral patterns. Therefore, further studies are necessary to explore the relationships among occupational stress and its domains, work shifts, and weight gain among female workers. Understanding the causal links between these health conditions is vital for developing effective preventive strategies for this population.

## Data Availability

The raw data supporting the conclusions of this article will be made available by the authors, without undue reservation.
